# *ODAPH* (p.Arg77*) Phenotype and Onset of Ameloblast Pathology During Postsecretory Transition Demonstrated by FIB-SEM Analyses of *Odaph*^C41*/C41*^ Mice

**DOI:** 10.1007/s00223-026-01562-2

**Published:** 2026-07-03

**Authors:** Jan C.-C. Hu, Charles E. Smith, Hong Zhang, Yuanyuan Hu, Tian Liang, Shih-Kai Wang, Kuan Yu Chu, Evans H. Brown, James P. Simmer

**Affiliations:** 1 Department of Biologic and Materials Sciences & Prosthodontics, University of Michigan School of Dentistry, 1011 N University Ave, Ann Arbor, MI 48109 USA; 2 Department of Anatomy & Cell Biology, Faculty of Medicine and Health Sciences, McGill University, Montreal, QC Canada; 3 Department of Orthodontics and Pediatric Dentistry, University of Michigan School of Dentistry, Ann Arbor, MI USA; 4 Department of Dentistry, National Taiwan University School of Dentistry, No.1, Changde St., Taipei City 100, Taiwan; 5 Department of Pediatric Dentistry, National Taiwan University Children’s Hospital, No.8, Zhongshan S. Rd., Taipei City 100, Taiwan

**Keywords:** Amelogenesis imperfecta, Enamel, Maturation, Adhesion, Cyst

## Abstract

Defects in *Odontogenesis-Associated Phosphoprotein* (*ODAPH*) cause autosomal recessive non-syndromic amelogenesis imperfecta (AI; OMIM #614832). We present a 14-year-old boy with delayed tooth eruption and generalized soft and yellow enamel. The proband’s parents and two siblings were unaffected. Whole exome analyses identified the AI-causing homozygous *ODAPH* truncation mutation (p.Arg77*) in the proband. Both unaffected parents and one sibling were heterozygous for this change. The other sibling had the reference sequence. To better understand the role of ODAPH in normal amelogenesis and resolve conflicting reports of ODAPH expression and function during postsecretory transition (PST), we performed comprehensive Focused-Ion Beam Scanning Electron Microscopy analyses of continuously growing mandibular incisors from one wild-type and two *Odaph*^C41*/C41*^ mice. Montages of sagittal sections were constructed using 5000 × images covering the surface enamel, ameloblasts, stratum intermedium and papillary layer during late secretory, PST, and early maturation stages. *Odaph*^C41*/C41*^ ameloblasts completed the secretory stage showing no evidence of pathology. Dramatic histological differences between wild-type and *Odaph*^C41*/C41*^ ameloblasts were observed during postsecretory transition closely preceding the onset of cyst formation in *Odaph*^C41*/C41*^ ameloblasts. PST ameloblasts show marked abnormalities, including loss of polarity (nucleus moves distally), disrupted cellular organization, indistinct cell boundaries and rapid reduction in cell height. During PST and early maturation, a dark line indicative of a basal lamina is apparent in the superficial enamel, but it fails to maintain attachment with the ameloblast distal membrane, and a cyst forms between the enamel surface and the overlying flattened ameloblasts, which eliminates the entire maturation stage of amelogenesis. Immunohistochemistry of a Day 12 mandibular incisor detected ODAPH protein in PST and the maturation stage ameloloblasts, and concentrated along the basement membrane. We conclude that ODAPH is required for a successful transition into the maturation stage of amelogenesis. Loss of ODAPH function results in hypomineralized amelogenesis imperfecta.

**Supplementary Information:**

The online version contains supplementary material available at 10.1007/s00223-026-01562-2.

## Introduction

*Odontogenesis-Associated Phosphoprotein* [*ODAPH* (OMIM *614829)] is a tooth-specific gene originally designated as *C4orf26* that, when defective, causes non-syndromic amelogenesis imperfecta (AI) in humans [1]. Dental enamel formation is a multistage process carried out by specialized epithelial cells called ameloblasts. Secretory stage ameloblasts initiate enamel mineral ribbons on the surface of freshly mineralized dentin and lengthen the ribbons along the secretory portion of their distal membrane [2–4], often described as the “mineralization front”. As the ameloblast distal membrane retreats, it expands the enamel layer by lengthening the enamel ribbons to achieve its final thickness and shape. Secretory ameloblasts then undergo a brief postsecretory transition (PST) that accomplishes a major cellular reconfiguration [5] into maturation stage ameloblasts that harden the enamel layer as they modulate between ruffled- and smooth-ended morphologies [6]. Only about 14% of total mineral by volume in rat incisor enamel is acquired by the time the maturation stage begins [6]. During the maturation stage the thin mineral ribbons, which extend uninterrupted from the dentin-enamel junction to the enamel surface, grow exclusively in width and thickness and become interlocked with adjacent crystals. The mineral added during enamel maturation occurs at the expense of matrix proteins and enamel fluid, without increasing the total volume of the enamel layer [7].

Mutations in more than 100 genes are associated with inherited enamel defects [8]. Most AI conditions are syndromic, meaning the enamel malformations are only one of multiple phenotypes likely to affect the patient. Isolated or non-syndromic forms of amelogenesis imperfecta are not accompanied by a non-dental phenotype [9]. *ODAPH* mutations are categorized as causing hypocalcified or hypomaturation, non-syndromic AI based upon the enamel radiographic appearance of unerupted teeth, as the soft enamel undergoes rapid and extensive attrition following eruption. *ODAPH* mutations are also associated with delayed tooth eruption [10], a phenotype that is sometimes observed in patients with inherited enamel defects [11]. To date, only seven unique AI-causing mutations in *ODAPH* have been reported [10]. Most reported AI-causing *ODAPH* mutations are truncations (Table 1).

**Table 1:** Published *ODAPH* mutations causing AI. Only seven disease-causing mutations have been reported. The cDNA variants are listed for the reference sequence transcript variant 2 (TV2), which predominates in human dental tissue [10]

#	Gene (NG_032974.1)	TV2 (NM_178497.5)	Protein	References
1	g.5074_5081del	c.39_46del	p.(Cys14Glyfs*21)	[12]
2	g.5086_5091delinsATGCTGGTTACTGGTA	c.51_56delinsATGCTGGTTACTGGTA	p.(Val18Cysfs*23)	[1]
3	g.5103del	c.67 + 1del	p.(Gly23Aspfs*140)	[10]
4	g.13065A > T	c.68-2A > T	p.(?)	[1]
5	g.13128C > A	c.129C > A	p.(Cys43*)	[1]
6	g.13228C > T	c.229C > T	p.(Arg77*)	[1] (Here)
7	g.13317G > A	c.318G > A	p.(Trp106*)	[1]

The apparent tooth-specific phenotype in patients with variants in *ODAPH* is strongly supported by the consistent finding that the *Odaph* gene is degenerated in vertebrates that have lost the ability to make teeth during evolution [13–15]. Previously we demonstrated that mouse *Odaph* mRNA is specifically expressed by ameloblasts starting at the onset of postsecretory transition and diminishes midway through the maturation stage of amelogenesis [16]. *Odaph* is not expressed by ameloblasts during the preceding secretory stage of amelogenesis, so the final thickness of the enamel layer is not disturbed in *Odaph* knockout mice [16, 17]. There is apparent selection against *Odaph* expression during the secretory stage, as artificial early expression of *Odaph* during the secretory stage results in dysmorphology of secretory stage ameloblasts and failure of normal appositional enamel deposition [18]. ODAPH is a constituent of the specialized basal lamina attaching maturation ameloblasts to the enamel surface [17, 19]. *Odaph* knockout mice show that failure of enamel maturation is consistently associated with the formation of cysts between the enamel surface and the flattened ameloblasts lining the cyst [16, 17]. In the literature, there is agreement that *Odaph* is expressed by early maturation stage ameloblasts, but importantly, one group demonstrates the onset of *Odaph* expression during postsecretory transition by in situ hybridization (PST) [16] while another observed no *Odaph* expression during PST using immunohistochemistry [17]. Recently, *ODAPH* transcripts have also been amplified from human dental pulp [10], although no dentin malformations have been reported in humans or *Odaph* null mice. This finding is intriguing as selection for *Odaph* is tooth-specific, but not enamel-specific [13]. A necessary role in dentinogenesis might explain the observed selection for *Odaph* in animals that retain enameless teeth but not in animals that have lost the ability to make teeth altogether.

To better understand how the absence of functional ODAPH protein causes the observed amelogenesis imperfecta phenotype, we further characterize *Odaph*^C41*/C41*^ knockout mice [16]. This mouse expresses a premature translation termination signal homologous to a human *ODAPH* truncation that causes autosomal recessive amelogenesis imperfecta in humans [1]. If translated, only the 22 amino acid signal peptide and N-terminal 18 amino acids of the secreted protein would be synthesized in *Odaph*^C41*/C41*^ mice. (The signal peptide is cleaved and degraded intracellularly prior to secretion of the protein.) Mice heterozygous for the p.Cys41* truncation are indistinguishable from the wild-type (*WT*) in phenotype, indicating no detectable dominant negative effects are caused by the truncated peptide [16]. We use focused ion beam-scanning electron microscopy (FIB-SEM) to visualize and compare the ultrastructure of the surface enamel, ameloblasts, stratum intermedium, and the papillary layer in the interval from late secretory stage, through postsecretory transition, and into early maturation in a *WT* (*Odaph*^+/+^) and two *Odaph*^C41*/C41*^ 7-week mandibular mouse incisors.

## Materials and Methods

### Human Subjects

The study protocol (H03-00001835-M1) and subject consent forms were reviewed and approved by the Institutional Review Board of the University of Michigan. Informed consent was obtained from all participants and/or their legal guardians. Study explanation, pedigree construction, subject enrollment, clinical examinations, and collection of saliva samples were completed under the proper consenting procedure specified in the study protocol and according to the Declaration of Helsinki.

### Laboratory Animals

The animal study protocol, #PRO00012569, was reviewed and approved by the Institutional Animal Care and Use Committee at the University of Michigan and complied with US National Research Council's Guide for the Care and Use of Laboratory Animals [20], and the US Public Health Service's Policy on Humane Care and Use of Laboratory Animals. All experiments were performed in accordance with relevant guidelines and regulations, and the authors complied with the ARRIVE (Animal Research: Reporting of In Vivo Experiments) guidelines. Mice were housed in a pathogen-free environment and maintained on a regulated light-cycle and standard rodent chow. The sample size was determined based on prior studies and relevant literature. In this study, we present one *WT* (from a pool of 50 + mice processed for FIB-SEM imaging) and two *Odaph*^C41*/C41*^ mandibular incisors (from a pool of 3 mice processed for FIB-SEM imaging).

### Genomic DNA Characterization and Analysis

Unstimulated saliva samples (2 ml) were collected from each recruited family member. Genomic DNA was extracted from each saliva sample using the prepIT•L2P gDNA isolation solution (Norgen Biotek Corp). Genomic DNA quality was evaluated by electrophoresis on a 1.5% agarose gel and quantified with a Qubit Fluorometer (Thermo Fisher Scientific). Genomic DNA from the proband was characterized by whole exome sequencing at the Johns Hopkins Center for Inherited Disease Research (CIDR). Variant annotation and mutational analyses were conducted using established protocols [21]. The homozygous *ODAPH* variant was determined during an initial screening for genetic variations within a list of candidate genes associated with hereditary enamel defects [22] and confirmed by Sanger sequencing.

### Segregation Analyses using Sanger Sequencing

PCR amplification of *ODAPH* exon 2 followed by Sanger sequencing for recruited family members was performed (Fig. S1) to determine segregation of the identified sequence variant with the disease phenotype. The PCR primers (F: 5'TCATTGAACGTTGGCAGTTC; R: 5'TCCAATTTTGCAACCATCAA) were used to generate a 675 bp amplification product. Each PCR reaction contained 20 µL of Platinum Hot Start PCR Master Mix (2x) (Invitrogen, Carlsbad, CA, USA), 2 µL of 10 µM primer mix, 2 µL of DNA template (final conc. < 500 ng/rxn) and raised to 40 µL with distilled water. The reactions were run using a GeneAmp PCR System 9700 Thermocycler (Applied Biosystems, Foster City, CA, USA). PCR conditions were 94 °C for 2 min, then 35 cycles of 94 °C for 30 s, then 58 °C for 30 s followed by 72 °C for 50 s, 72 °C for 2 min and then hold at 4 °C. PCR products were purified using QIAquick PCR Purification Kit (Qiagen, Hilden, Germany) and sent for Sanger sequencing at the U-M Advanced Genomics Core.

### Immunohistochemistry

Rabbit anti-mouse ODAPH polyclonal antibodies were generated against modified synthetic peptides corresponding to mouse ODAPH residues 114–126: C-Ahx-QQ-pS-G-pS-pS-pS-EE-pS-REN-COOH. The peptide was conjugated to KLH, phosphorylated at Ser116, Ser118, Ser119, Ser120, and Ser123, and HPLC-purified prior to immunization. The resulting antiserum was affinity-purified, and the antibody titer was determined by ELISA (YenZym Antibodies LLC, Brisbane, CA, USA). Hemi-mandibles from D12 wild-type C57BL/6 mice were dissected and fixed overnight at 4 °C in 4% paraformaldehyde (PFA) prepared in phosphate-buffered saline (PBS). Samples were subsequently decalcified in 16.52% disodium ethylenediaminetetraacetic acid (EDTA, pH 7.4) at 4 °C with rotation for 14 days. Following decalcification, samples were dehydrated through an ethanol gradient, cleared in xylene, embedded in paraffin, and sectioned at 5 μm thickness using a HistoCore AUTOCUT R microtome (Leica, Deer Park, IL, USA). Sections were mounted onto Fisherbrand™ Tissue Path Superfrost Plus Gold microscope slides (Thermo Fisher Scientific, Waltham, MA, USA) and processed for immunohistochemistry according to established protocols [23]. Affinity-purified rabbit anti-mouse ODAPH residues 114–126 polyclonal antibodies (YenZym Antibodies LLC) were used as primary antibodies at a dilution of 1:100. Alexa Fluor™ Plus 594 goat anti-rabbit IgG (H + L) secondary antibody (1:1500 dilution; A32740, Invitrogen) was used for detection. Nuclei were counterstained with DAPI (P36931, Invitrogen), and sections were mounted using ProLong™ Diamond Antifade Mountant (Thermo Fisher Scientific, Waltham, MA, USA). Images were acquired using an EVOS™ M7000 Imaging System (Thermo Fisher Scientific) for low-magnification imaging and a Nikon A1R confocal microscope (Nikon, Melville, NY, USA) for high-magnification imaging.

### Focused Ion Beam Scanning Electron Microscopy (FIB‑SEM)

Hemimandibles from 3 wild-type and 3 *Odaph*^C41*/C41^ (C57BL/6N-Odaphem1Jcch/Mmnc MMRRC:067431-UNC) 7-week-old mice that had been fixed by vascular perfusion with a weak glutaraldehyde solution were removed, washed in buffer and embedded in epoxy plastic as described previously [22, 24]. After polymerization, the incisors were partitioned with a saw into sequential 1-mm-thick transverse sections. These were mounted flat with the most incisal side facing upward on plastic stubs with superglue. The third cross section in a tooth series (designated as Level 3) usually contained the region of postsecretory transition. These were cut away from their stubs, rotated upwards at 90° and remounted with superglue on a second stub. This changed the orientation for sectioning from the transverse (cross) to sagittal (longitudinal) plane relative to the incisor. The enamel and enamel organ area of interest were faced into proper orientation with glass knives. In preparation for FIB-SEM imaging, 3 wild-type and 2 *Odaph*^C41*/C41^ blocks were sawed away from their subs and attached to 45° chamfered metal microscope mounting stubs with conductive silver paste. The stubs were placed in the imaging chamber of a Helios Nanolab 660 FIB-SEM (FEI, Systems for Research, Longueuil, QC, Canada). A sampling area 100 μm × 100 μm in size was selected and milled with gallium ions at rough (45 nÅ) followed by fine (9.4 nÅ) settings. Imaging was performed at various magnifications from × 1,500 to up to × 35,000 with the through lens detector (TLD) and where possible with the in-column detector (ICD). Surface charging on block faces was reduced by coating them with a thin layer of platinum (3 nm) where required.

## Results

**Fig. 1:**
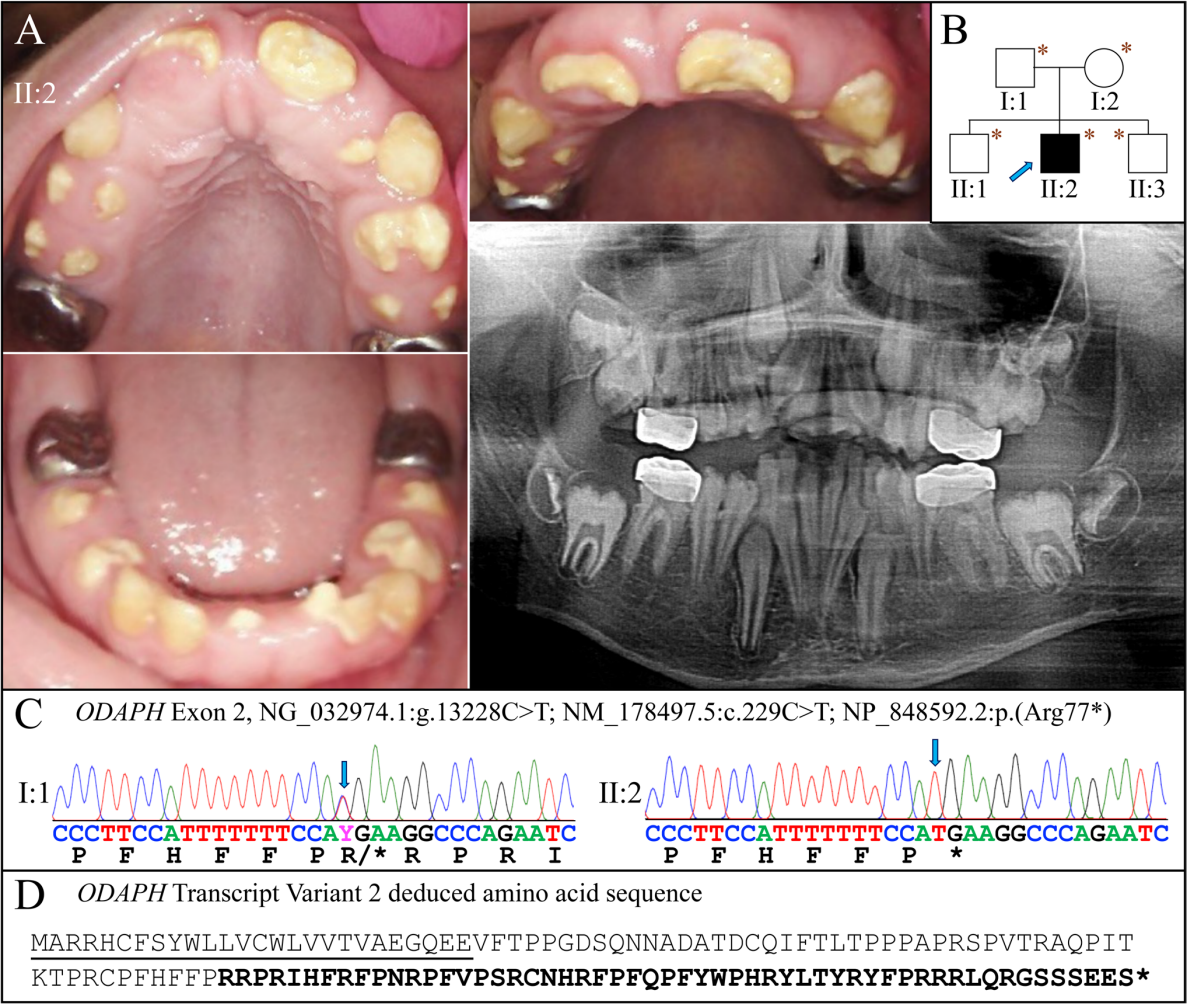
Hypomaturation Amelogenesis Imperfecta Caused by an *ODAPH* Homozygous Truncation Mutation (p.Arg77*). **A** Oral phenotype and panoramic radiograph of the male proband (II:2) at age 12.5 years. The permanent dentition is not fully erupted (delayed). The gingiva still partially covered the maxillary bicuspids. All four second molars failed to erupt, with the mandibular molars showing a small coronal radiolucency with a thin sclerotic border, indicating they had not yet emerged from the overlying alveolar bone. The enamel of unerupted teeth was slightly more radiopaque than dentin and underwent rapid attrition following eruption. **B** Both parents (I:1 and I:2) and the younger brother (II:3) were heterozygous for the same *ODAPH* variant (c.229C > T) that converted arginine codon 77 (CGA) into a STOP (TGA) codon. *Denotes study participants. Only the proband showed a detectable enamel phenotype. **C** DNA sequence chromatograms showing the father’s heterozygous *ODAPH* sequence and the proband’s (II:2) homozygous sequence for the variant that caused the AI phenotype (Table 1). **D** Amino acid sequence of *ODAPH* transcript variant 2 (NM_178497.5). The signal peptide that is cleaved after directing the protein into the secretory pathway is underlined and the amino acids lost due premature termination of translation are in bold

The proband was a healthy 14-year-old-boy with an unremarkable medical history except for an emergency visit due to a painful episode of gallbladder stone inflammation and obstruction. Parents reported that the patient’s permanent first molars were covered with stainless steel crowns at age 11.0 years to prevent further attrition and to maintain vertical dimension. His oral and radiographic presentations at age 12 years 6 months are shown in Fig. 1, along with the family pedigree and sequence analyses characterizing the homozygous *ODAPH* mutation that caused his enamel phenotype (Suppl. Fig. S1). A few days after the photos and panoramic radiograph were taken, the maxillary and mandibular primary teeth (including 4 canines and 4 lateral incisors) were removed to facilitate the eruption of his underlying delayed lateral incisors and canines. None of the second molars had yet erupted. The mandibular second molars were not impacted against the first molars, but had not erupted through the alveolar bone, as indicated by a thin radiolucent line observed over the pericoronal space on radiographs (Fig. 1).

Whole exome analyses identified a sequence variant in both alleles of the proband’s *ODAPH* gene that introduced a premature translation termination codon. The GRCH38 reference sequence designations for this *ODAPH* variant are NC_000004.11:g.76489485C > T; NC_000004.12 (NM_178497.5):c.229C > T; NM_178497.5 (NP_848592.2):p.(Arg77*). Both parents (I:1 and I:2) and the younger brother (II:3) were heterozygous for this *ODAPH* variant (c.229C > T) that converted arginine codon 77 (CGA) into a translation STOP codon (TGA). Sequence chromatograms of all participants are shown in Suppl. Fig. S1. All heterozygous family members for this mutation showed normal enamel, suggesting that the dental phenotype of the homozygous proband was likely caused by a loss of function rather than a gain of function or dominant negative mechanism. The cDNA and protein designations corresponding to transcript variant 2 (TV2), which predominates during mouse tooth development [10] and is the most conserved *Odaph* splice variant among therians. *Odaph* is a therian neomorph only found in marsupials and eutherians but is not detected in the monotreme platypus [13].

Currently only 7 AI-causing mutations in *ODAPH* have been reported, all showing an autosomal recessive pattern of inheritance (Table 1). Each of these seven reported AI-causing mutations in *ODAPH* is predicted to either truncate the protein product or to induce nonsense mediated decay of the mutated mRNA transcript.

### Ameloblast Ultrastructure Centering on Postsecretory Transition

To better define the onset of this pathology and clarify the nature of ODAPH’s role in amelogenesis, we prepared sagittal sections of *Odaph*^+/+^ and *Odaph*^C41*/C41*^ mouse mandibular incisors and imaged them using FIB-SEM. Overlapping 5000 × micrographs were collected and assembled across successive ~ 100-µm-wide sagittal preparations, or “runs”. Each run captured the surface enamel, ameloblasts, and papillary layer generating a continuous ultrastructural record of the late secretory stage, postsecretory transition (PST), and early maturation stage from one *Odaph*^+/+^ and two *Odaph*^C41*/C41*^ mouse mandibular incisor sagittal sections. The run numbers are preserved in the figures and figure legends as records to ensure data tracking and accuracy.

**Fig. 2:**
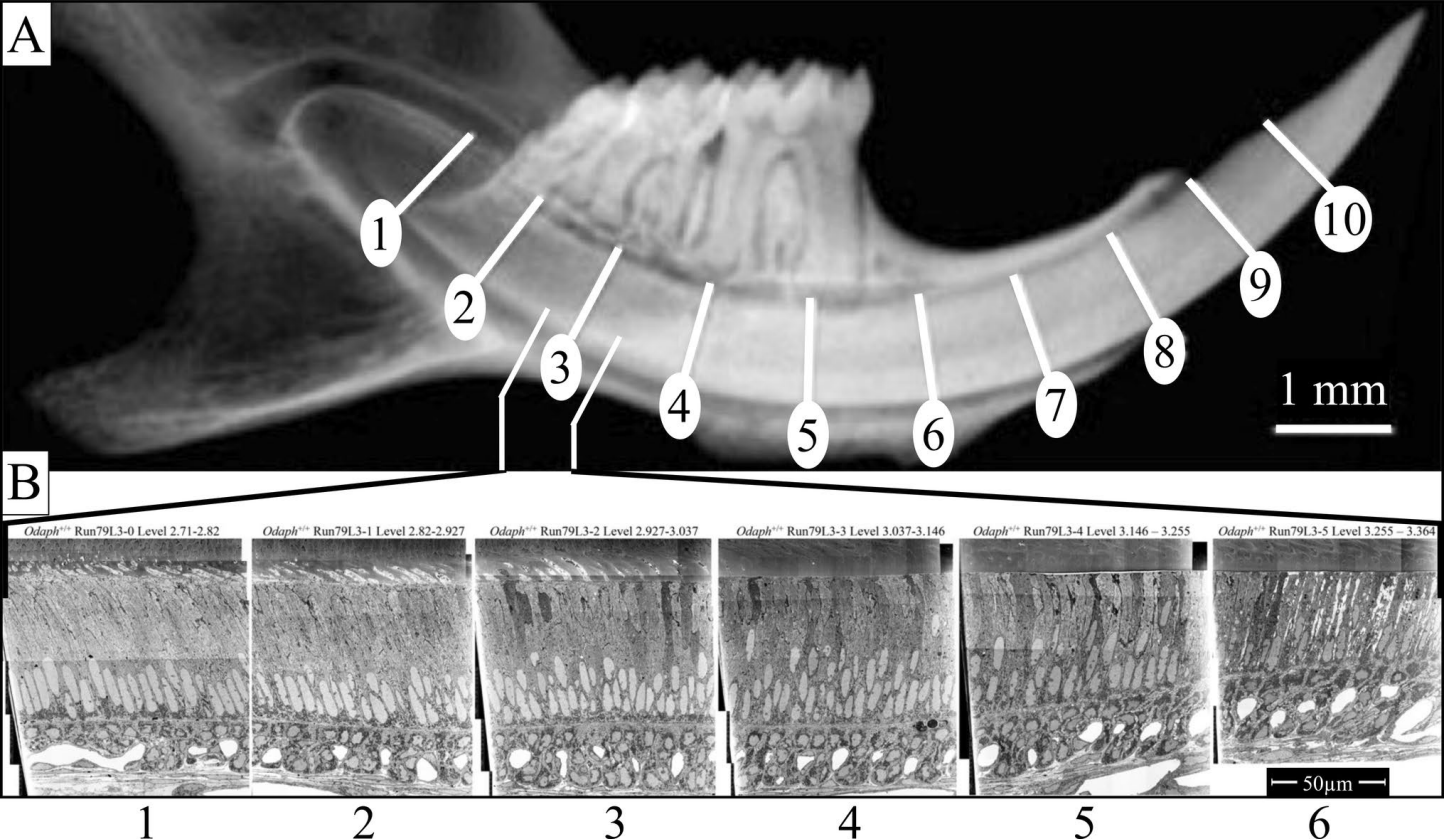
X-ray image of hemimandible showing wild-type molars and incisor **A** and wild-type histological montages of 5000 × FIB-SEM micrographs **B** showing late secretory stage ameloblasts (Panels 1–3) and their transition into maturation stage ameloblasts (Panels 4–6). *A:* Radiograph of a wild-type (*WT*) mandible at ~ 7 weeks. The 10 slices through the incisor show the standard locations where mandibular incisors are routinely cross-sectioned at 1 mm increments and characterized by bSEM imaging, where the progression of the secretory stage of amelogenesis is assessed in cross-sections 1 through 3and the maturation stage in cross-sections 3 through 9. This is typically done to compare *WT* to genetically altered mice (single gene knockouts). In this study we generated six montages of longitudinal images (cut in a sagittal plain) covering the end of the secretory stage (starting at level 2.71), postsecretory transition, and early maturation stage up to level 3.36. Brackets on either side of the #3 delineate the 0.65 mm linear distance of the sagittal section analyzed by FIB-bSEM imagine. In the 7 weeks mandibular incisor, Level 0 starts at the tip of the apical loop and travels along the base of the epithelium in a curvilinear path that becomes linear well before the onset of dentin and enamel biomineralization. For practical (dissection) purposes, the white bone lining the apical socket is 0 mm. One mm incisal to this is Level 1.0, which marks the onset of enamel biomineralization. Level 3.0 marks the border at the end of the secretory stage and the onset of postsecretory transition. *B:* Six panels comprised of consecutive, longitudinally-sectioned *WT* incisor montages (called “runs) are aligned showing the enamel at the top, above the underlying tall columnar ameloblasts and papillary layer at the bottom. These six consecutive longitudinal sections span the brackets on both sides of #3. In Fig. S3 we show the block that was used to obtain these images. The subsequent supplemental figures (S4-S8) show pairs of these panels at higher magnification. This analysis centers on postsecretory transition where ODAPH expression and ameloblast pathology first appear. This image is a larger version of Fig. 2A, shown here to provide context for the segments of mandibular incisors that were imaged, evaluated, and presented in Figures S4-S8

**Fig. 3:**
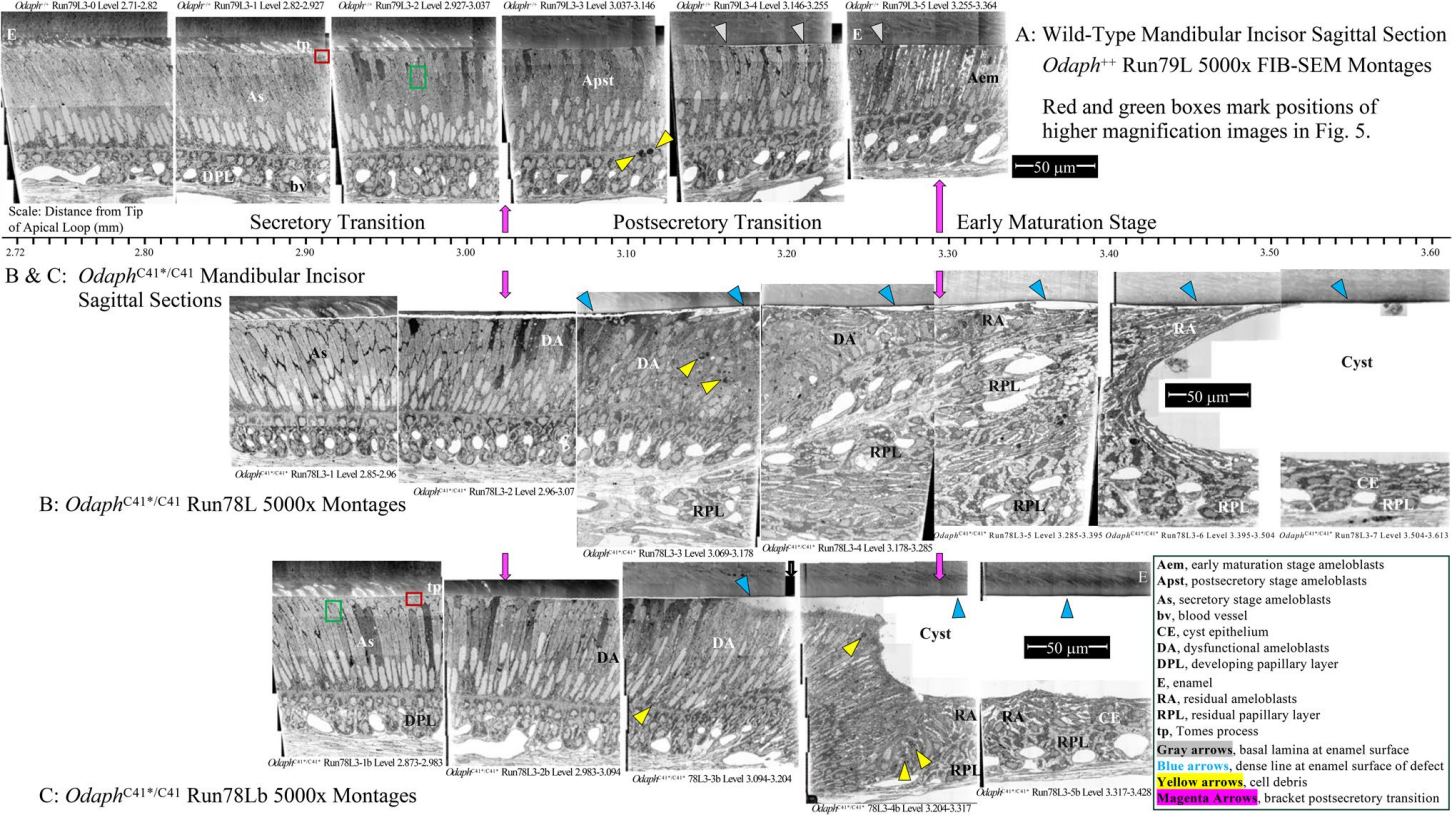
FIB-SEM 5000 × montages comparing successive mandibular incisor sagittal sections from 7-week **A** *Odaph*^+/+^ and (**B** & **C**) *Odaph*^C41*/C41*^ mice. The montages are oriented (from top to bottom) showing the enamel, ameloblast, and papillary layers. The aligned sections start with ameloblasts in late secretory stage (left). Postsecretory transition (center) is bracketed with magenta arrows. Early maturation stage is on the right. The scale below the wild-type montages provides the location on the incisor, with 0 at the tip of the apical loop and ~ 1.0 mm at the onset of enamel mineralization (not shown). Postsecretory transition typically starts at ~ 3.0 mm and ends with modulating ameloblasts of reduce height ~ 3.3 mm. A key for abbreviations is provided on the lower right. Colored boxes mark the positions of higher magnification images shown in Fig. 5. *Key:* Aem, early maturation stage ameloblasts; As, secretory ameloblasts; Apst, postsecretory transition stage ameloblasts; bv, blood vessel; CE, cyst epithelium; DA, dysfunctional ameloblasts; DPL, developing papillary layer; E, enamel; RA, residual ameloblast; RPL, residual papillary area; tp, Tomes’ process. Blue arrowheads show a dense line at the defective enamel surface in *Odaph*^C41*/C41*^ incisors. Gray arrowheads mark the wild-type maturation stage basal lamina. Yellow arrowheads mark cell debris. Magenta arrows bracket PST

Mouse incisors are continuously growing and display all the developmental stages of amelogenesis (dental enamel formation) sequentially, with earliest development apically (to the left) and later development incisally (to the right). The position of the area of interest (end of secretory stage, postsecretory transition, and beginning of maturation stage) on the mouse mandibular incisor at 7-weeks is provided in Fig. 2. Montages assembled from overlapping 5000 × images of an *Odaph*^+/+^ and two *Odaph*^C41*/C41*^ mouse mandibular incisors are presented in Fig. 3.

All three sagittal sections presented in this comparison begin in late secretory stage when ameloblasts are retracting their Tomes’ process as it extends the enamel mineral ribbons to the final surface of the incisor enamel layer. This is followed by postsecretory transition (PST), a brief period of cellular reorganization needed to redirect the ameloblasts’ activities from elongating the enamel mineral ribbons (expanding the enamel layer) to thickening and widening them, which hardens the existing enamel layer. As the ameloblasts proceed through postsecretory transition, they reduce in height and emerge as shorter, maturation stage ameloblasts that modulate between ruffled- and smooth-ended morphologies. Suppl. Fig. S2 shows the wild-type block face with outlines of the consecutive positions of the 6 *WT* runs used to produce the montaged *WT* FIB-SEM images, which are shown at the bottom.

Figure 3 shows the full extent of the FIB-SEM characterization of the *Odaph*^+/+^ and two *Odaph*^C41*/C41*^ incisor sagittal sections aligned to permit relevant comparisons. We also include extensive supplemental figures to provide enlarged and higher magnification images of the Fig. 3 montages, and their context in the 7-week-old mandibular incisor. Suppl. Figs. S3 through S7 show larger versions of the six *WT* FIB-SEM runs presented in Fig. 3A, along with higher magnification details. Suppl. Figs. S8 through S11 show larger versions of the *Odaph*^C41*/C41*^ montages presented in Fig. 3B. Suppl. Figs. S12 through S17 show larger and higher magnification versions of the montages from the second *Odaph*^C41*/C41*^ mouse presented in Fig. 3C.

In Fig. 3, magenta arrows bracket postsecretory transition, which immediately succeeds the secretory stage that is completed by retraction of the ameloblast Tomes’ processes as they extend the rod enamel ribbons to the final enamel surface, so ameloblasts rest on a smooth enamel surface when they enter postsecretory transition. These clearly visible changes and a transition from an incisal to apical tilt of the ameloblasts ensure precise synchronization of the onset of PST.

### Late Secretory Stage Ameloblast Ultrastructure

Micrographs of *Odaph*^+/+^ (Fig. 3A; Suppl. Figs. S2-S5) and *Odaph*^C41*/C41*^ (Figs. 3B, C and Suppl. Figs. S8-S9, S12-S13) incisors clearly demonstrate incisally-tilted secretory stage ameloblasts in the process of retracting their Tomes’ processes (TP) as they deposit the final enamel layer. In both *Odaph*^+/+^ (Fig. 3A and Suppl. Figs. S6, S7) and *Odaph*^C41*/C41*^ mice (Fig. 3B, C and Suppl. Figs. S10-S11, S15-S16) the Tomes’ process retracts as ameloblasts complete the final extension of enamel mineral ribbons to fill in the space formerly occupied by the TP which establishes a smooth enamel surface closely associated with the now flattened ameloblast distal membrane that completed formation of the final enamel layer immediately prior to the onset of postsecretory transition.

**Fig. 4:**
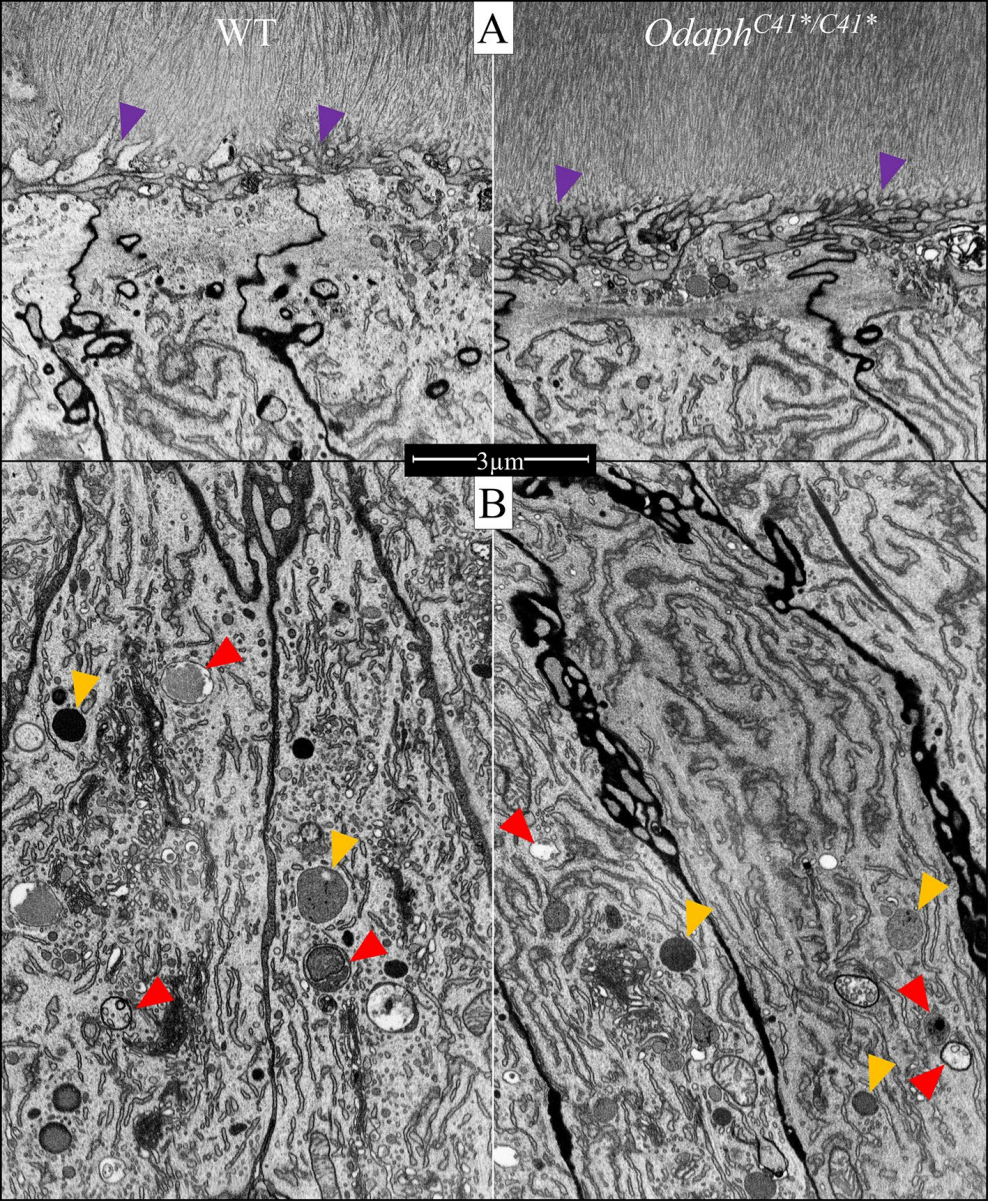
Wild-type micrographs from *WT*54 Run79L3-1 (left) compared to *Odaph*^C41*/C41*^ Run78L3-1b. **A** This area, in both incisors nearing the end of secretory stage, shows the typical appearance of incisally-tilted ameloblasts containing highly developed Golgi and lysosomal structures. Both wild-type and *Odaph*^C41*/C41*^ ameloblasts show transient microvillar structures, or ruffling (purple arrowheads), along their distal surfaces as the final enamel layer is applied to the enamel surface. The positions of these 20,000 × images are outlined by red boxes in the Fig. 3 montages. **B** Wild-type micrograph (*left*) showing ameloblasts from *WT*54 Run79L3-2 are compared to *Odaph*^C41*/C41*^ Run78L3-1b (*right*). Late secretory stage micrographs of *Odaph*^C41*/C41*^ and wild-type late secretory stage incisor ameloblasts show comparable lysosomes (gold arrowheads), enlarged and denser intercellular spaces, degradative organelles and autophagic vacuoles (red arrowheads). The positions of these 10,000 × images are outlined by green boxes in the Fig. 3 montages

Figure 4 compares the ultrastructure of *Odaph*^+/+^ ameloblasts near the end of secretory stage. At higher (20000x) magnification, late secretory stage ameloblasts from both *Odaph*^+/+^ and *Odaph*^C41*/C41*^ incisor ameloblasts (Fig. 4A) show transient microvillar structures, or “ruffling” along their distal membrane as the final enamel layer is applied to the enamel surface. Similarly, *Odaph*^+/+^ and *Odaph*^C41*/C41*^ incisors (Fig. 4B) show highly developed Golgi and lysosomal structures with no evidence of pathology. The ultrastructural histology of late secretory stage ameloblasts is indistinguishable between *Odaph*^+/+^ and *Odaph*^C41*/C41*^ incisors. This strongly supports the conclusion that ODAPH function is not necessary during the secretory stage of amelogenesis.

### Ameloblast Ultrastructure During Postsecretory Transition

**Fig. 5:**
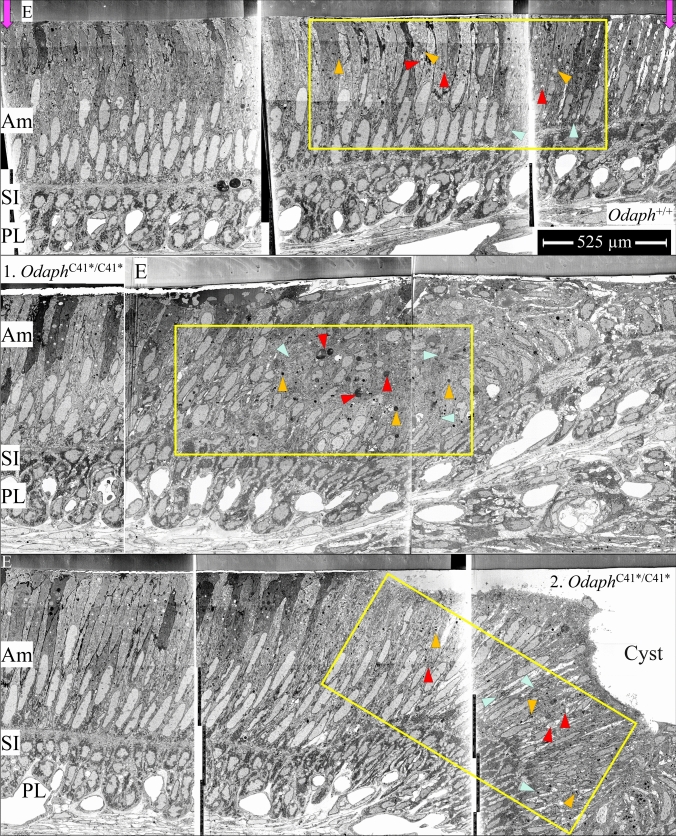
Postsecretory transition stage ameloblasts in *Odaph*^+/+^ and *Odaph*^C41*/C41*^ mandibular incisors. These montages of 5000 × FIB-SEM images specifically compare *WT* (top) and two *Odaph*^C41*/C41*^ incisors (middle and bottom) starting when there is a smooth ameloblast distal membrane against a flattened enamel surface. PST ends after PST ameloblasts have progressively reduced in height to the size of maturation stage ameloblasts (magenta arrow on right). In the *Odaph*^+/+^ sample (top), PST cells show a steady decrease in ameloblast cell height characteristic of PST. Magenta arrows delineate PST and are in the same position as in Fig. 3. On the far left, at the onset of PST, the ameloblasts from the three incisors are comparable and no longer lean incisally, as they did immediately earlier during late secretory stage (Fig. 3). Dramatic differences between the *WT* and *Odaph*^C41*/C41*^ incisors develop during PST. Yellow boxes outline regions of obvious cell pathology. *Key:* E, enamel; Am, ameloblast; PL, papillary layer; SI, stratum intermedium; arrowheads: gold, lysosomes; red: phagosomes, light blue: shrunken nuclei)

The onset of postsecretory transition immediately follows the disappearance of Tomes’ processes and the filling-in with mineral of the spaces they occupied to achieve a smooth ameloblast distal membrane against a flattened surface of enamel. Postsecretory transition ends after PST ameloblasts complete their transition from secretory to maturation ameloblasts after steadily shrinking in cell height to that of maturation stage ameloblasts that modulate between smooth and ruffled-ended forms to accomplish the maturation (hardening) of enamel. To accurately compare PST in the *WT* and *Odaph*^C41*/C41*^ incisors, we previously aligned the three FIB-SEM montages to synchronize the onset of PST when constructing Fig. 3. We enlarged this region to better compare the *WT* and two *Odaph*^C41*/C41*^ incisors specifically in the PST region (Fig. 5).

Following completion of the secretory stage, ameloblasts express a novel attachment apparatus that includes AMTN [25, 26], ODAM [27], LAMC2 [28] SCPPPQ1 [29]. The new basement membrane proteins start to accumulate at the ameloblast-enamel interface during post-secretory transition [27], but the attachment is initially relatively weak and even wild-type PST ameloblasts tend to undergo artifactual detachment from the enamel surface during histological preparation [5]. In Fig. 5, artifactual separation of ameloblasts during PST is apparent in all three mice, but is more severe in the *Odaph*^C41*/C41*^ incisors where PST ameloblasts start showing marked abnormalities, including loss of cell polarity (nucleus moves distally), disrupted cellular organization, indistinct cell boundaries, and a rapid reduction in cell height. The pathology in both *Odaph*^C41*/C41*^ incisors during PST is unmistakable but varies. In the first *Odaph*^C41*/C41*^ incisor (Figs. 3B and 5 middle) the cyst does not start until the *WT* incisor is in early maturation stage, while the second *Odaph*^C41*/C41*^ incisor (Figs. 3C and 5 bottom) initiates cyst formation.

### ODAPH Expression During Amelogenesis

**Fig. 6:**
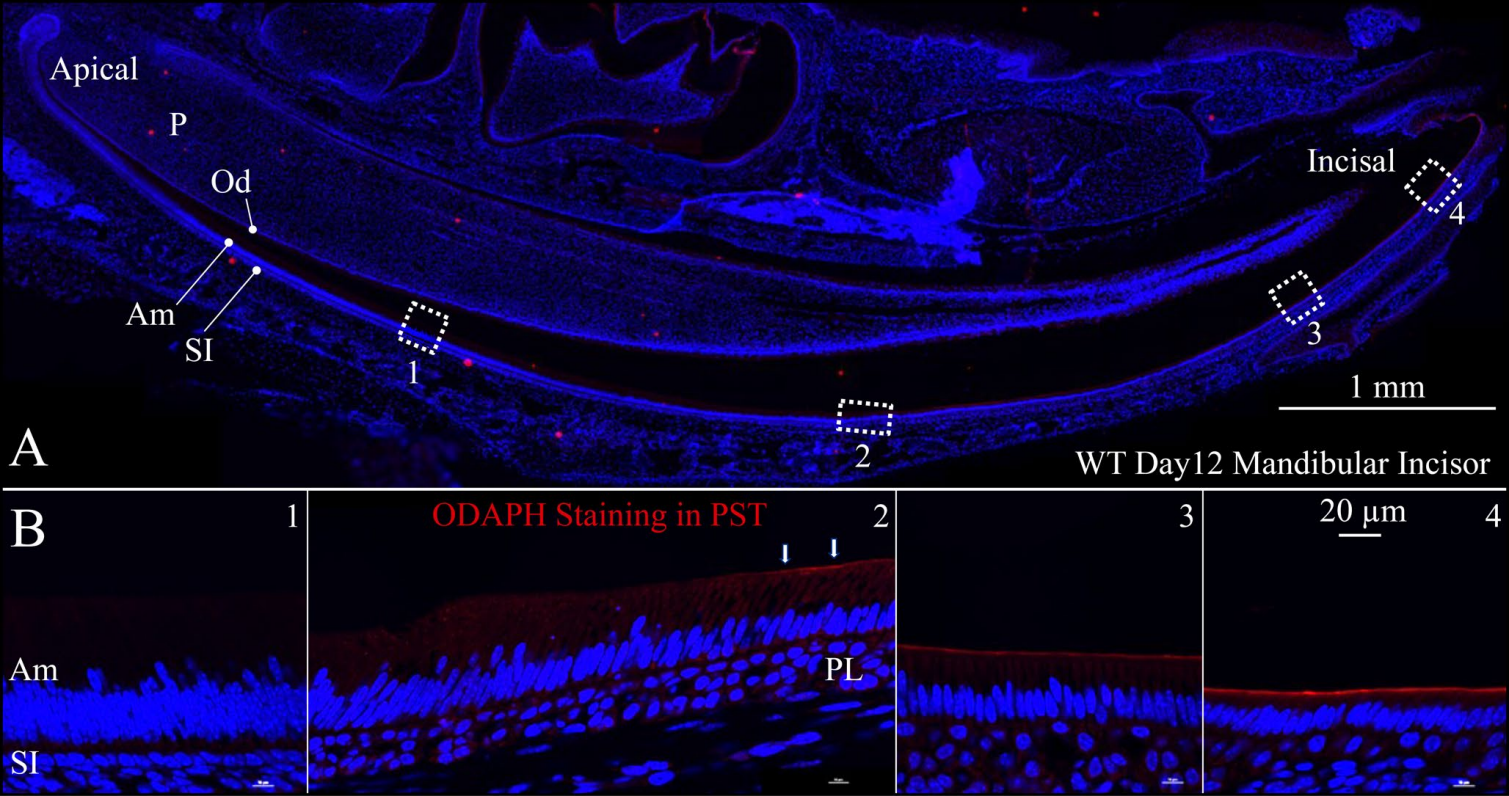
Immunohistochemistry of ODAPH expression in a *WT* postnatal day 12 mouse mandibular incisor. **A** Immunostained incisor sagittal section with 4 dashed boxes outlining the positions of the 4 higher magnification images displayed in the second panel. **B** No ODAPH immunostaining was detected in secretory-stage ameloblasts (panel 1). Positive ODAPH immunostaining was first detected in transition stage ameloblasts (panel 2), which are recognized by their position in the incisor, their initial apical tilt (ameloblast nuclei tilt towards the apical side of the incisor), and their progressively diminishing cell height. Initially the ODAPH staining is intracellular but concentrates in the newly synthesized basement membrane (white arrows). Positive ODAPH immunostaining persists in the ameloblast basement membrane throughout the subsequent maturation stage (panels 3 and 4). Key: Am, ameloblast; Od, odontoblast; P, pulp; PL, papillary layer; SI, stratum intermedium

To clarify its timing of expression during amelogenesis, a phosphorylated peptide containing mouse ODAPH residues 114–126 was synthesized to raise antibodies and to affinity purify them. These antibodies were used to immunostain a day 12 mandibular incisor sagittal section, which displays progressive changes in ameloblast activity in a linear array (Fig. 6).

## Discussion

### ODAPH Tooth Specificity, Human Mutations, and Dental Phenotype

When assessing a patient with inherited dental defects it is important to determine if the condition is isolated (confined to the dentition) or syndromic (has or will show defects in other tissues). Over 100 different genes are involved in the etiology of genetic conditions feature inherited enamel malformations (amelogenesis imperfecta) and over 90% of these conditions include malformations in other tissues. As the dental phenotype is often the first observed, dentists must refer patients with inherited dental malformations to a clinical geneticist to order genetic tests to identify the specific mutation causing their condition. Taking this prudent step to obtain a genetic diagnosis may allow early interventions to mitigate the severity of systemic conditions and the dental treatment may qualify for payment under medical insurance or other programs.

Confidence that variants in a particular gene are not associated with systemic abnormalities comes from studying other vertebrates that have lost the ability to make teeth or specifically dental enamel during evolution. The *Odaph* gene is inactivated in mammals that have lost the ability to make teeth during evolution, including the pangolin, bowhead whale, two minke whale species and eight baleen whale species [13, 15]. In addition, *Odaph* remains functional in three mammalian species that retain teeth, but have independently lost the ability to make dental enamel [13]. The only other gene known to share this pattern is dentin sialophosphoprotein (*Dspp*) [13]. Based upon similar studies, eight genes (*Acp4*, *Ambn*, *Amelx*, *Amtn*, *Enam*, *Klk4*, *Mmp20*, and *Odam*) are known to be functionally enamel-specific, becoming inactivated during evolution in vertebrates that have lost the ability to form dental enamel [13, 15, 30, 31]. Finding a causative genetic variant in one of the other 100 + genes associated with inherited enamel defects is likely to be syndromic, and the patient’s physician is contacted and informed to ensure proper management of the syndrome.

To date, only 7 AI-causing mutations in *ODAPH* have been published (Table 1). All are homozygous variants following an autosomal recessive pattern of inheritance. The seminal first report was in 2012, at a time when the *ODAPH* gene was still designated as *C4orf26* [1]. They identified 5 homozygous *ODAPH* mutations in 9 families with “autosomal recessive hypomineralized AI”. No patient ages or panoramic radiographs were presented, and there was no mention of delayed or failed tooth eruption. The second *ODAPH* mutation report provided intraoral photographs and panoramic radiographs of three affected individuals, an adult and two children in the primary dentition stage with “hypomineralized-hypoplastic AI” [12]. The third report described a proband with “autosomal recessive hypocalcified AI” [10]. The panoramic radiograph revealed that teeth #6, 7, 8, 9, 10, 11, 22, and 27 had failed to erupt and there was no discernable dentinoenamel junction (DEJ) of these unerupted teeth (as the dentin and enamel layers exhibited similar radiodensities). They also noted that *ODAPH* mRNA could be amplified from the dental pulps of developing and exfoliating primary teeth. This surprising finding may explain the observation that there is selection pressure to maintain a functional ODAPH gene/protein in organism that makes teeth containing dentin, but no enamel.

### The ODAPH Protein and Gene, and Relationship to Other Genes and Proteins

ODAPH is an acidic phosphoprotein containing ten possible phosphorylation sites [1], including multiple Golgi casein kinase (GCK) recognition motifs [32]. GCK is encoded by *FAM20C* [33], which phosphorylates secreted proteins containing Ser-x-Glu/phospho-Ser (SxE/pS) motifs. This motif is a characteristic feature of the secretory calcium binding phosphoprotein (SCPP) family [34], which includes many proteins associated with tooth mineralization, such as AMELX, ENAM, AMBN, AMTN, ODAM, and DSPP. While ODAPH is not evolutionarily related to SCPP proteins, its gene localizes between the 2 major groups of SCPP gene clusters on human chromosome 4 and is also phosphorylated by GCK.

Golgi casein kinase (FAM20C) and its interaction with FAM20A is required to efficiently phosphorylate enamel proteins in the secretory pathway, so mutations in either one of these genes cause severe enamel malformations [35, 36]. FAM20C functions as the catalytic kinase and becomes fully activated through homodimerization or heterodimerization with FAM20A [37], but FAM20C alone carries the kinase activity. Loss-of-function mutations in either gene result in severe enamel defects and are frequently associated with delayed, failed, or ectopic tooth eruption [36]. Mutations in *FAM20A* (OMIM *611062) cause enamel-renal syndrome, AI type IG (OMIM #204690) [38–40]. *FAM20C* (OMIM *611061) variants cause Raine syndrome (OMIM #259775), which is typically lethal [36]. When non-lethal, it shows a generalized skeletal osteosclerosis [41]. Rarely, individuals with Raine Syndrome survive into old age [42].

The phenotypes observed in patients with Enamel Renal Syndrome and Raine Syndrome reflect the combined loss of phosphorylation of all GCK-dependent secreted proteins. Mutations in SCPP genes that disrupt their GCK motifs have been shown to cause enamel defects [43, 44]. While failure to phosphorylate secreted enamel matrix proteins, namely AMELX, ENAM, and AMBN likely account for the enamel defects in Enamel Renal Syndrome and Raine Syndrome, the mechanism underlying impaired tooth eruption remains unclear. The finding that inactivating mutations in *ODAPH* (a protein with multiple GCK phosphorylation sites) also cause a tooth eruption failure phenotype suggests that insufficient phosphorylation of ODAPH may contribute to the eruption defects observed in *FAM20A* and *FAM20C* disorders.

### ODAPH’s Role in Amelogenesis

Previously we showed that *Odaph* is specifically expressed by ameloblasts starting at the onset of postsecretory transition and continues through mid-maturation. SEM analyses of mandibular incisor cross-sections demonstrated that the enamel layer in *Odaph*^C41*/C41*^ mice reaches normal full thickness by the end of the secretory stage, comparable to the wild-type controls [16]. Using FIB-SEM to achieve higher magnification, we show that *Odaph*^C41*/C41*^ mice display normal ameloblast histology at the end of the secretory stage that is indistinguishable from *Odaph*^+/+^ mice. The pathology in *Odaph*^C41*/C41*^ ameloblasts begins and rapidly progresses during postsecretory transition. The dark line at the ameloblast distal membrane/enamel surface has historically been called the basal lamina or basal lamina-like structure and is known to contain AMTN [25], LAMC2 and ODAPH [17, 45], ODAM [27, 46], and SCPPPQ1 [29]. Our findings suggest that a loss of ODAPH does not prevent secretion of these components, as the dark line of the basal lamina remains visible in *Odaph*^C41*/C41*^ mice. However, without ODAPH the basal lamina increasingly fails to maintain ameloblast adhesion to the enamel surface as PST ameloblasts detach, lose polarity and develop significant cell pathology ending in cyst formation and catastrophic failure of enamel maturation.

In this study we present only the fourth report of an *ODAPH* mutation causing enamel defects in humans and present patient radiographs displaying enamel crowns with a full-thickness of enamel, but with diminished radiopacity and delayed eruption. Using the *Odaph*^C41*/C41*^ mouse model and employing focused ion beam-scanning electron microscopy (FIB-SEM) to generate montages that visualize the ultrastructure of the surface enamel and overlying enamel organ epithelia (ameloblasts, stratum intermedium, and papillary layer) spanning the interval from late secretory stage–postsecretory transition–early maturation in 7 weeks mandibular incisors of one *Odaph*^+/+^ and two *Odaph*^C41*/C41*^ mouse mandibular incisors to clearly demonstrate the onset of severe ameloblast pathology during postsecretory transition that leads to catastrophic cyst formation between the enamel and flattened ameloblasts, which blocks maturation (hardening) of the full-thickness enamel layer deposited normally during the secretory stage. In addition, we confirm the onset of ODAPH expression in PST ameloblasts and its specific localization in late PST and the maturation stage basement membrane. These findings are consistent with and explain the human phenotype of an enamel layer of normal thickness (prior to eruption into function) that is poorly mineralized due to the failure of enamel maturation following postsecretory transition.

## Supplementary Information

Below is the link to the electronic supplementary material.

Supplementary file1

## Data Availability

The datasets presented in this study can be found in online repositories. The human subject dataset “Genetics of Disorders Affecting Tooth Structure, Number, Morphology and Eruption” can be browsed at dbGaP: https://www.ncbi.nlm.nih.gov/projects/gap/cgi-bin/study.cgi?study_id=phs001491.v4.p1 The mouse strain, MMRRC_067431-UNC (C57BL/6N-Odaph^em1Jcch^/Mmnc), is available at the Mutant Mouse Resources & Research Centers. Study data of *Odaph*^C41*/C41*^ are available at FaceBase dataset 1-MCT2 entitled “Effects of *Odaph* Gene Knock-out on Mouse Tooth Development.
