# Supplementary material for: ODAPH (p.Arg77*) Phenotype and Onset of Ameloblast Pathology During Postsecretory Transition Demonstrated by FIB-SEM Analyses of OdaphC41*/C41* Mice
Source: Calcif Tissue Int. 2026 Jul 3;117(1):107. doi: 10.1007/s00223-026-01562-2 (PMC13331816; doi:10.1007/s00223-026-01562-2)
Supplement: Supplementary file 1 — Supplementary file1 [file 223_2026_1562_MOESM1_ESM.docx]

***ODAPH* (p.Arg77*) Phenotype and Onset of Ameloblast Pathology During Postsecretory Transition Demonstrated by FIB-SEM Analyses of *Odaph*^C41*/C41*^ Mice**

Jan C.-C. Hu^1*^, Charles E. Smith^1,2*^, Hong Zhang^1^, Yuanyuan Hu^3^, Tian Liang^3^,

Shih-Kai Wang^4,5^, Kuan Yu Chu^1^, Evans Brown^1^, James P. Simmer^1**^

^1^Department of Biologic and Materials Sciences, University of Michigan School of Dentistry, Ann Arbor, MI, USA

^2^Department of Anatomy & Cell Biology, Faculty of Medicine and Health Sciences, McGill University, Montreal, Quebec, Canada

^3^Department of Orthodontics and Pediatric Dentistry, University of Michigan School of Dentistry, Ann Arbor, MI, USA

^4^Department of Dentistry, National Taiwan University School of Dentistry, No.1, Changde St., Taipei City 100, Taiwan.

^5^Department of Pediatric Dentistry, National Taiwan University Children’s Hospital, No.8, Zhongshan S. Rd., Taipei City 100, Taiwan.

*Jan C.-C. Hu and Charles E. Smith are co-first authors

**Email Addresses:**	janhu@umich.edu; charles.smith@mcgill.ca; zhanghon@umich.edu; tianl@umich.edu; yyhu@umich.edu; shihkaiw@ntu.edu.tw; [kychu@umich.edu](mailto:kychu@umich.edu); evansbro@umich.edu; [jsimmer@umich.edu](mailto:jsimmer@umich.edu)

**List of Supplementary Materials**

Figure S1.	Chromatograms of *ODAPH* transcript variant 2 from the parents and three children.

Figure S2.	Tissue block used to produce the wild-type FIB-SEM micrographs.

Figure S3.	Wild-type ameloblasts forming the outer enamel at the end of secretory stage.

Figure S4.	Montages of high magnification WT images to demonstrate specific details of Fig. S3.

Figure S5.	WT ameloblasts forming outer enamel at Levels 2.927-3.146.

Figure S6.	Montages of high magnification WT images to demonstrate specific details of Figure S5.

Figure S7.	WT ameloblasts in postsecretory transition at Levels 3.146-3.364.

Figure S8.	Seven Run78L 5000x montages of FIB-SEM micrographs showing *Odaph*^C41*/C41*^ (null mouse #1) mandibular incisor enamel forming cells.

Figure S9.	*Odaph*^C41*/C41*^ ameloblasts completing the secretory stage and entering postsecretory transition (Levels 2.82-3.069).

Figure S10.	*Odaph*^C41*/C41*^ ameloblasts degenerating during postsecretory transition (PST) Levels 3.069-3.285.

Figure S11.	*Odaph*^C41*/C41*^ PST ameloblasts continued degeneration into squamous cell lining a cyst (Levels 3.285-3.613).

Figure S12.	Montages of 5000x FIB-SEM micrographs showing *Odaph*^C41*/C41*^ (null mouse #2) pathology developing in transition stage ameloblasts followed by cyst formation.

Figure S13.	*Odaph*^C41*/C41*^ ameloblasts forming the outer enamel (Levels 2.873-2.983).

Figure S14.	*Odaph*^C41*/C41*^ ameloblasts have completed the outer enamel and are now in postsecretory transition (Levels 2.983-3.094).

Figure S15.	*Odaph*^C41*/C41*^ ameloblasts in transition stage (Levels 3.094-3.204) degenerating into a cyst.

Figure S16.	*Odaph*^C41*/C41*^ ameloblasts in transition stage (Levels 3.204-3.317) degenerating into a cyst.

Figure S17.	*Odaph*^C41*/C41*^ ameloblasts (Levels 3.317-3.428) are reduced to squamous cells lining a cyst.

**Figure S1. Chromatograms of *ODAPH* transcript variant 2 from the parents and three children.** The mutation designated as NM178497.5: c.229C>T; NP_848592.2: p.(Arg77*) resulted in a premature stop codon at amino acid position 77 (blue arrow). Both parents (I1 and I2) and the youngest sibling (II3) are heterozygous for the variant. The oldest child (II1) has two reference alleles. The proband (II2) has two mutant alleles. Only the proband presented with hypomaturation amelogenesis imperfecta and delayed tooth eruption.

**Figure S2. Tissue block used to produce the wild-type FIB-SEM micrographs.** *Top:* FIB-SEM of block face. *Bottom:* Light microscopy of a 0.50 µm thick semi-thin section stained with toluidine blue. Red boxes outline the six longitudinal runs imaged by FIB-SEM and are presented as 5000x montages (Fig. 2). For comparison, similar 7-week mandibular incisors blocks were prepared for two *Odaph*^C41*/C41*^ mice and then imaged by FIB-SEM. ***Key:*** Am, ameloblasts; D, dentin; E, enamel.

**Figure S3. Wild-type ameloblasts forming the outer enamel at the end of secretory stage.**

***Left:*** The proximal ends of incisor ameloblasts (Am) forming the outer enamel typically lean incisally (ameloblast nuclei tilt toward the tip of the incisor). Enamel (E) is at the top of the image. Apically (toward the apical loop) is leftward. Incisally (toward the incisal tip) is rightward. The ameloblast end (Tomes’ process) embedded in the enamel is distal, while the end adjacent to the stratum intermedium and papillary layer (pl) is proximal. This set of images is typical of the secretory stage ameloblasts and developing papillary layer (pl). The ameloblasts show very large Golgi areas and are loaded with lysosomes of various sizes and contents. Some ameloblasts show ragged clusters of dark material in a supra and infra nuclear location which we suspect are glycogen masses. Mitochondria in ameloblasts vary greatly in shape and cell position (supranuclear v. proximal; ameloblast v. papillary cells).

***Right:*** Late secretory stage mandibular incisor ameloblasts are still forming outer (final) enamel and lean incisally. Enamel mineral ribbons continue to elongate near the distal cell membrane as the Tomes’ processes retract to form a smooth enamel surface. The four box outlines mark the locations of higher magnification images in Fig. S4.

**Figure S4. Montages of high magnification WT images to demonstrate specific details of Fig. S3.** *A:* The ER and Golgi areas are highly developed, and the cells are characteristically fully loaded with a variety of lysosomal structures, which can be small or large, and dense or pale. *B:* Changes are starting to happen in ameloblasts including the beginnings of a shift in location of some mitochondria from the proximal end toward the supranuclear compartment. *C:* Alterations in the proximal region on the papillary layer side of the proximal terminal web, which is very prominent. Microvilli seem to be increasing in the intercellular spaces of the papillary layer.

**Figure S5.** **WT ameloblasts forming outer enamel at Levels 2.927-3.146.** Enamel appositional growth ends at Level 3.0 with the onset of postsecretory transition (PST). The incisor enamel reaches its final thickness, which is ~121µm from DEJ to the enamel surface at its thickest point. The distal end of the secretory stage ameloblast is marked by the disappearance of the Tomes’ process protruding into the enamel layer. **Panel A *(Left):*** A few ameloblasts at the most incisal side (on the right) are in PST, as evidenced by the flattened distal surface with slight undulations. Ameloblasts incisal to this point are a mixture of those completing enamel formation and those already in PST. Mineral still abuts against the ameloblast distal membranes (no lamina densa) in early PST. Mitochondria in these cells are on the move around the nucleus to enter the supranuclear compartment. As this happens, they become less dense and the pattern of cristae inside them changes. The ameloblasts remain rich in lysosomes but autophagic vacuoles are added to the mix of large intracellular lysosomal structures. Golgi areas are still well developed. The ameloblasts are oriented vertically or angled slightly apically rather than incisally as they were during formation of the outer enamel layer. The proximal end of ameloblasts shows highly developed tight junctions, which appear as dense lines on the proximal membrane with filaments of the terminal web running between them. The papillary layer is starting to increase in height. **Panel B *(Right):*** Across this area, the ameloblasts are starting to shrink in height. They are angled more toward the apical loop than in previous areas. Ameloblasts at the incisal side of this area are loaded with autophagic vacuoles helping to dispose of excess Golgi, ER and mitochondria. There are large clusters of cell debris (cd) in the former stratum intermedium indicative of some cell death. There is no evidence in this sample of invasion by macrophages. Papillary layer cells are presumably removing the debris resulting from the reduction in number and size of ameloblasts. The distal and proximal tight junctions are still well-developed, but changes are evident along the proximal ends of ameloblasts where cell debris is present. There is no evidence of a basal lamina (dark line) at the enamel surface where crystallites abut against the ameloblast distal membrane at the incisal side of this area early in PST. The two boxed areas at the cell-enamel surface are shown at higher magnification in Fig. S6.

**Figure S6. Montages of high magnification WT images to demonstrate specific details of Figure S5.** The top panel is the 5000x montage of the enamel layer of WT Run79L3-3. Below is the 20000x montage of the area near the lower far right of the top panel showing a basal lamina has not yet assembled even though ameloblasts are clearly losing height following the onset of postsecretory transition. The onset of PST is recognized by the disappearance of Tomes’ processes and a flattened, smooth profile of the ameloblast distal membrane against the enamel surface.

**Figure S7. WT ameloblasts in postsecretory transition at Levels 3.146-3.364.** In the latter half of PST, ameloblasts continue to reduce in cell height, organelle content/distribution, and junctional arrangements (most of the left panel). The basal lamina starts to coalesce at the enamel surface as a dense structure seemingly “sinking into” the superficial enamel (into the enamel surface as opposed to lying on top of it). Autophagic vacuoles are not observed after the end of PST (right panel). The modulating ameloblasts start accumulating a plethora of pale and dense lysosomes around the Golgi region. Development of the papillary layer looks complete. The ameloblasts in the right panel look smooth-ended with enlarged intercellular spaces, but the presence of distal tight junctions suggests these cells may be transforming toward ruffle-ended if we were to progress more incisally from the panel on the right. The intracellular contents of ameloblasts are typical of early modulation. The papillary layer is fully developed. ***Key:*** E, enamel; AM, ameloblast; pl, papillary layer.

**Figure S8. Seven Run78L 5000x montages of FIB-SEM micrographs showing *Odaph*^C41*/C41*^ (null mouse #1) mandibular incisor enamel-forming cells.** On the left, ameloblasts are completing the secretory stage of enamel formation (panel 1 and part of panel 2). The secretory stage ameloblasts enter postsecretory transition and undergo rapid degeneration in panels 3 and 4, reducing into a thinner cell layer that continues to collapse into squamous cells that detach from the enamel layer to line the inner surface of a cyst, resulting in no maturation of the enamel layer. These panels will be shown in pairs in the following figures and explained further. This image is a larger version of Fig. 3B, shown here to provide context for the segments of mandibular incisors that were imaged, evaluated, and presented in Figs. S10-S12.

**Figure S9. *Odaph*^C41*/C41*^ ameloblasts completing the secretory stage and entering post-secretory transition (Levels 2.82-3.069).** The ameloblasts at this site look normal for the most part. Their organelle content includes a highly developed Golgi apparatus and numerous lysosomes. The Tomes’ processes but show typical thin and elongated shapes angled sharply in an apical direction. Whether the space been the distal end of the ameloblasts and the enamel surface is a weakness related to this genotype, or a tearing artifact is unclear. The LM image of this block face suggests it might be an extension of loss of surface adhesion that become much more pronounced in the next imaging field as we enter postsecretory transition. The stratum intermedium remains well-defined, and the stellate reticulum has developed into cuboidal-shaped cells of the outer dental epithelium. Vascular invaginations typical of the papillary layer are in development. Only a few ameloblasts on the right of 78L3-2 are in PST, as evidenced by the flattened distal surface with slight undulations. Ameloblasts up to this point are a mixture of those completing enamel formation and those within PST. Mineral still buts the distal membranes of cells in early PST. Mitochondria in these cells are on the move around the nucleus to enter the supranuclear compartment, and as this happens, they become less dense and the pattern of cristae inside them changes. The ameloblasts remain rich in lysosomes but autophagic vacuoles are added to the mix of large intracellular lysosomal structures. Golgi areas are still well developed. The ameloblasts are positioned more vertical or angled slightly apically rather than angled incisally, as during secretion of the outer enamel layer. The proximal end of ameloblasts shows highly developed tight junctions which appear as dense lines on the proximal membrane with filaments of the terminal web running between them. The papillary layer is starting to increase in height.

**Figure S10.** ***Odaph*^C41*/C41*^ ameloblasts degenerating during postsecretory transition (PST) Levels 3.069-3.285.** *Left:* The change from tall ameloblast into a confusing mess of shrinking and deteriorating cells occurred rapidly (happening within hours; in sagittal sections, 1 hour of cell time = ~10 µm of apical-incisal length). The distal end of ameloblasts has some blobs of cytoplasm embedded in a pool of homogeneous protein. All ameloblasts are overloaded with degradative organelles. The papillary layer becomes more disorganized, and the cells change from polygonal to a more flattened shape. Fibroblasts in the labial CT are plump and full of well-developed organelles. Former ameloblasts are flattened or polygonal in shape and still show signs deterioration and disorganization. The former papillary layer continues to show some usual organizational features but is noticeably shrunken in thickness. At high magnification some cells in the residual enamel organ and adjacent connective tissue show surprisingly well-developed Golgi areas, and some cells at the middle and apical side of the block face still show typical secretory granules and evidence of lateral secretion of proteins into the intercellular spaces. Some of the vascular sinuses in the deteriorating papillary layer show clear signs of being fenestrated (very thin membranes stretching across pore areas in the plasma membrane).

**Figure S11.** ***Odaph*^C41*/C41*^ PST ameloblasts continued degeneration into squamous cell lining a cyst (Levels 3.285-3.613).** The papillary layer is pretty much the only recognizable feature of the former enamel organ. Blood vessels outside the papillary layer (teal arrowheads) look pretty much normal. The intercellular space in the papillary layer remains loaded with microvilli. Outer connective tissue is now comprised of sheets of fibroblasts. Flattened cells line the walls of the cyst.

**Figure S12. Montages of 5000x FIB-SEM micrographs showing *Odaph*^C41*/C41*^ (null mouse #2) pathology developing in transition stage ameloblasts followed by cyst formation.** In panel 1 on the left, the proximal ends of late secretory stage ameloblasts tilt incisally. These ameloblasts are retracting their Tomes’ process as they extend the enamel mineral ribbons to establish the smooth, slightly undulating “final enamel” surface. In panel 2 the ameloblasts have entered postsecretory transition. Note that the enamel surface is no longer penetrated by Tomes’ processes and the proximal ends of ameloblasts have shifted their tilt apically. The onset of pathology in *Odaph* null transition stage ameloblasts occurs in panel 2 and rapidly increases in severity in panel 3 resulting in detachment of ameloblasts from the enamel surface in the formation of a large cyst (panels 4 – 5), with ameloblasts being reduced to squamous cells lining the cyst. As a result, no enamel maturation, or hardening of the enamel layer by thickening the fully elongated thin mineral ribbons that were extended to the final enamel surface during the secretory stage. The five individual montages assembled into this figure are shown individually in the following figures, along with 3 thin vertical strips of these runs imaged at 10000x (sometimes two of the strips merged so only 2 strips are presented). Magnifications showing details from these runs are also presented and explained. This image is a larger version of Fig. 3C, shown here to provide context for the segments of mandibular incisors that were imaged, evaluated, and presented in Figs. S14-S18.

**Figure S13.** ***Odaph*^C41*/C41*^ ameloblasts forming the outer enamel (Run Levels 2.873-2.983).** The panel on the left is the first run from null mouse #2. The panel on the right shows vertical strips taken at higher magnification. *Odaph* null ameloblasts show the typical appearance of outer enamel formation with the ameloblast proximal end (containing the nucleus) tips angled incisally and the cells contain highly developed Golgi and lysosomal structures. These late secretory stage images are like those of the wild-type, as they precede the onset of pathology that occurs during PST. Compare these images to wild-type Figs. S4 and S5. The secretory stage ameloblasts look normal and successfully elongate enamel mineral ribbons to achieve a normal thickness of enamel. Their organelle contents include a highly developed Golgi apparatus and numerous lysosomes. The Tomes’ processes show typical thin and elongated shape angled sharply in an apical direction. The stratum intermedium remains well defined, and the stellate reticulum has developed into cuboidal shaped cells as the outer dental epithelium. Vascular invaginations typical of the papillary layer are in development. As in WT mice, the Tomes’ processes retract while continuing to elongate the enamel mineral ribbon to fill the surface depression they occupied and then deposit a thin layer of rodless enamel, after which enamel mineral ribbons are no longer elongated at the mineralization front and the full thickness of the enamel layer is achieved.

**Figure S14.** ***Odaph*^C41*/C41*^ ameloblasts have completed the outer enamel and are now in postsecretory transition (Levels 2.983-3.094).** *Odaph*^C41*/C41*^ ameloblasts have now transitioned from ameloblasts with proximal ends angled incisally to those angled apically. The supranuclear region of ameloblasts is loaded with numerous pale and dark lysosomes of various sizes and contents. Mitochondria in ameloblasts are on the move into the supranuclear compartment and the distal end of the cell. Golgi regions in both ameloblasts and papillary layer cells are enlarged. Ameloblasts show signs of variable and poor adherence to the enamel surface.

**Figure S15.** ***Odaph*^C41*/C41*^ ameloblasts in transition stage (Levels 3.094-3.204) degenerating into a cyst.** PST ameloblasts remain loaded with numerous pale and dark lysosomes of various sizes. Mitochondria in ameloblasts are on the move into the supranuclear compartment and the distal end of the cell. Ameloblasts are shrinking in height but do not look healthy (intercellular spaces are enlarging, the ER is prominent at the distal end (lime arrowheads). Tight junctions are disappearing from the distal ends of ameloblasts related to the defect forming at the incisal side of the block face. As in *Odaph*^C41*/C41*^ mouse 1 (Fig. S11) the change from tall ameloblast into a confusing mess of shrinking and deteriorating cells is very rapid (happening within hours; in sagittal sections, 1 hour of cell time = ~10 µm of apical-incisal length). The distal end of ameloblasts contains some blobs of cytoplasm embedded within a pool of homogeneous protein (lavender arrowheads). All ameloblasts are overloaded with degradative organelles. The papillary layer becomes more disorganized, and the cells change from polygonal to a more flattened shape.

**Figure S16. *Odaph*^C41*/C41*^ ameloblasts in transition stage (Levels 3.204-3.317) degenerating into a cyst.** The dark line on the surface of the enamel layer (5000x image only) is due to the presence of basement membrane components that have failed to adhere to the cells in the absence of ODAPH. Components of an ineffective basement membrane Former ameloblasts are flattened or polygonal in shape and still show signs deterioration and disorganization. The former papillary layer continues to show some usual organizational features but is noticeably shrunken in thickness. Some of the vascular sinuses in the deteriorating papillary layer show clear signs of being fenestrated (very thin membranes stretching across pore areas in the plasma membrane).

**Figure S17.** ***Odaph*^C41*/C41*^ ameloblasts (Levels 3.317-3.428) are reduced to squamous cells lining a cyst.** The cyst between the enamel layer and the cells is shown in Fig. 3C. Here, following cyst formation, the papillary layer is pretty much the only recognizable feature of the former enamel organ. Blood vessels outside the PL look pretty much normal. The intercellular space in the papillary layer remains loaded with microvilli (purple arrowheads). From these FIB-SEM studies it is established that defects in *Odaph* do not affect the secretory stage, which is able to achieve the final dimensions of the enamel layer. *Odaph* loss of function results in ameloblast degeneration and their transformation during the transition stage into simple non-ion transport type squamous epithelial cells that do not contribute to the maturation of the enamel layer, leaving the enamel hypomineralized and subject to failure during function.
